# The alkaloids of *Corydalis hendersonii* Hemsl. contribute to the cardioprotective effect against ischemic injury in mice by attenuating cardiomyocyte apoptosis via p38 MAPK signaling pathway

**DOI:** 10.1186/s13020-023-00726-8

**Published:** 2023-03-17

**Authors:** Fuxing Ge, Xiaoli Gao, Xiaochun Zhou, Junjun Li, Xiaojing Ma, Meiwen Huang, Sana Wuken, Pengfei Tu, Chao An, Xingyun Chai

**Affiliations:** 1 Modern Research Center for Traditional Chinese Medicine, Beijing Research Institute of Chinese Medicine, Beijing University of Chinese Medicine, Beijing, 102488 People’s Republic of China; 2 Dongfang Hospital, Beijing University of Chinese Medicine, Beijing, 100078 People’s Republic of China

**Keywords:** *Corydalis hendersonii* Hemsl., Tibetan folk medicine, Myocardial ischemia, Apoptosis, p38 MAPK signaling pathway, Alkaloids

## Abstract

**Background:**

There is a characteristic Tibetan folk medicine in China named *Corydalis hendersonii* Hemsl. (CH) has been used for treatment of cardiovascular related diseases, called “plethora” in Tibetan medicine. Previous studies demonstrated that ethanol extract of CH showed anti-acute myocardial infarction (AMI) effect through inhibiting fibrosis and inflammation. Rich alkaloids fraction (RAF) is isolated from CH, but whether RAF possessing an equivalent effect with the CH ethanol extract and by which mechanism it protects against AMI has not yet reported. The paper aimed to study the potential role of RAF on myocardial injured mice and its underlying mechanism.

**Materials and methods:**

Liquid chromatography mass spectrometry-ion trap-time of flight (LCMS-IT-TOF) was used to analyze the chemical profile and isolate pure compounds. The ligation of left anterior descending (LAD) of coronary artery in mice was used to evaluate the in vivo anti-AMI effect, by dividing into eight groups: Sham, Model, Fosinopril (10 mg/kg, i.g.), total extract (TE, 400 mg/kg, i.g.), poor alkaloids fraction  (PAF, 300 mg/kg, i.g.), and RAF (25, 50, and 100 mg/kg, respectively, i.g.) groups. Echocardiography was used to evaluate mice heart function through the index of left ventricular end-systolic  diameter (LVEDs), left ventricular end-diastolic diameter (LVEDd), fractional shortening (FS) and ejection fraction (EF). We detected the lactate dehydrogenase (LDH) and creatine kinase-MB (CK-MB) in the serum and the plasma level of angiotensin II (AngII). The apoptosis of mice myocardial tissue was verified by TUNEL assay. The expression of p38 mitogen-activated protein kinases (p38 MAPK), Bcl-2 and Bcl-2-associated X protein (Bax) were detected through immunofluorescence staining, qRT-PCR and western blot in mice heart tissue and H9c2 cells.

**Results:**

Echocardiography data indicated that the values of LVEDd and LVEDs were reduced and the values of FS and EF were improved by TE and RAF significantly. RAF also decreased the levels of LDH, CK-MB and AngII and significantly inhibited inflammatory cells in the marginal zone of myocardial infarction. The TUNEL assay results showed that RAF significantly attenuated cell apoptosis. Immunofluorescence and qRT-PCR assay showed that RAF inhibited p38 MAPK, Bax, and Bcl-2 proteins in mice myocardium. Western blot results validated that the expressions of key proteins were inhibited by RAF. Also, the apoptotic cells and apoptosis-related proteins were dramatically reduced by RAF in vivo and in vitro. Besides, RAF and PAF were analyzed by LCMS-IT-TOF to identify the main compounds and to demonstrate the difference between them. The results showed that a total of 14 alkaloids were identified, which indicated that the isoquinoline alkaloids were the main ingredients in RAF may contributing to the cardioprotective effect in mice.

**Conclusions:**

RAF improves cardiac function by inhibiting apoptosis via p38 MAPK signaling pathway, and RAF contributes to the effect against myocardial ischemic injury of TE in mice, which provides a substantial reference for the clinical application against ischemia heart disease and quality control of CH.

**Graphical Abstract:**

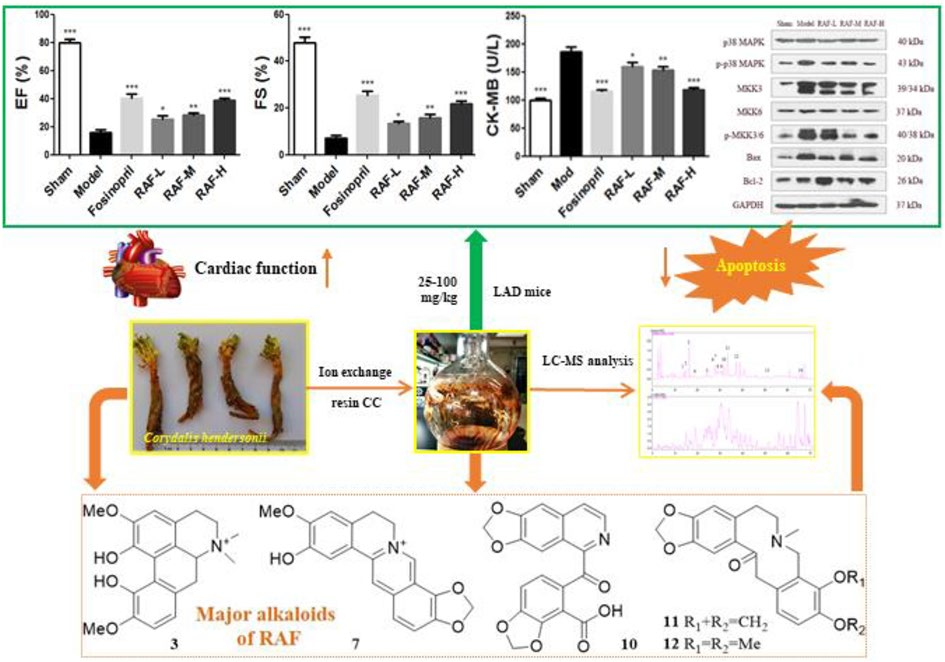

**Supplementary Information:**

The online version contains supplementary material available at 10.1186/s13020-023-00726-8.

## Highlights

- The total extract of *Corydalis hendersonii* Hemsl. (CH) and the rich alkaloids fraction (RAF) of CH showed significant cardioprotective effect on mice following acute myocardial infarction.
- RAF improved cardiac function by inhibiting apoptosis via p38 mitogen-activated protein kinases (p38 MAPK) signaling pathway in vitro and in vivo.
- The results of liquid chromatography mass spectrometry-ion trap-time of flight (LCMS-IT-TOF) showed that the 14 isoquinoline alkaloids in RAF as the main ingredients contribute to the cardioprotective effect.

**Supplementary Information:**

The online version contains supplementary material available at 10.1186/s13020-023-00726-8.

## Introduction

Ischemic heart disease (IHD), caused by the imbalance between coronary blood flow and myocardial demand due to changes in the coronary circulation, has a high incidence and fatality rate [1, 2]. Although the drug development and treatment methods in this area have been developed to a certain extent, the mortality rate of IHD has been rising rapidly since 2005, and the mortality rate of IHD is higher than that in the rural areas, and correspondingly the research and development of drugs for cardiovascular diseases treatment has been vigorously developed at home and abroad and seeking drugs for the treatment of IHD including from traditional medicine resources increasingly became one of hotspots in the aspects [3–5].

Our group has been focusing on ethnomedicines in China used to cure acute myocardial infarction (AMI)-related IHD, for instance the *Syringa pinnatifolia* Hemsl. [6–8], and *Corydalis hendersonii* Hemsl. (CH) and *Meconopsis horridula* Hook. f. & Thomson [9, 10].

 CH is one of the characteristic Tibetan folk medicine called “plethora” used to cure of high-altitude polycythemia (HAPC), hypertension and other diseases, which provides a new effective way for discovery of new therapeutic strategy against ischemic heart disease [11, 12]. Previously, the anti-AMI effect of ethanol total extract (TE) and underlying mechanism of anti-inflammation via JAK2-STAT3 and NF-κB signaling pathway has been investigated in our group [10]. However, whether alkaloid fraction exerts the anti-AMI effect equivalently with TE and whether it is underlying another mechanism need to further explore. In the present study, the effect and mechanisms of rich alkaloids fraction (RAF) against ischemic injury were evaluated by ligating the left anterior descending (LAD) coronary artery in ICR mice and H9c2 cells under hypoxic conditions.

## Materials and methods

### Preparation of TE, RAF and PAF

The whole plant of *Corydalis hendersonii* Hemsl. (the plant name has been checked with http://www.theplantlist.org) was collected from Shannan Autonomous Prefecture of Tibet in July 2017. Prof. Yuan Zhang (Beijing University of Chinese Medicine, BUCM) authenticated CH. The voucher specimen of CH was No. CH201707 and was stored in BUCM. About 160 g of total extract (TE) was obtained after extraction. Disperse the TE with hydrochloric acid aqueous solution and adjust the pH value to 2–3. Then the strong acid cation exchange resin is applied for separation and purification of alkaloids with the condition that passing through the hydrogen cation exchange resin column at the rate of 2 bed volumes per hour (BV/hour). The resin column was flushed with water at the rate of 2 BV/hour to neutral and the poor alkaloids fraction (PAF, 120 g) was obtained by collecting the combined eluent concentrated in vacuum. At last, the column was eluted at the rate of 2 BV/hour to obtain rich alkaloids fraction (RAF, 30 mg) with 90% alkaline ethanol whose pH value at 10–11.

### LCMS-IT-TOF

The research protocol was as the same with previous study [10]. The mobile phase (1.0 mL/min) consists of acetonitrile (A)–0.1% aqueous with formic acid (B) with a gradient program as follows: 0–15 min, 5%–15% A; 16–50 min, 15%–30% B; 51–80 min, 30%–95% B; Roughly 20% portion of the effluent was introduced into the ESI (source by splitting the effluent via two polyetheretherketone tubes with length ratio of 1:4). The diode array detector (DAD) detector scans its absorption wavelength in the full wavelength range of 190–400 nm, so we set a 10 μL injection volume and 254 nm detection wavelength.

### Experimental animal and ethics statement

The adult of male ICR mice (8 weeks, 27–28 g) were obtained from Sibei Fu Biotechnology Co., Ltd. (Beijing, China) and reared in a specific pathogen-free (SPF) environment. The mice were housed with regular food and free water in a 12 h light–dark cycles, under conditions of 22 ± 2 °C. According to the previous reports, the AMI model of mice LAD ligation was used to evaluate the effect of TE, RAF and PAF [9]. The Ethics Committee of BUCM had approved the experiments (the ethics permit number: BUCM-3-2015090701-3003), and the research was implemented according to the US guidelines (NIH publication #85–23, revised in 1996).

Eight groups were randomly divided: sham, model, Fosinopril (10 mg/kg, i.g.), TE (400 mg/kg, i.g.), PAF (300 mg/kg, i.g.), RAF high dose (RAF-H, 100 mg/kg, i.g.), RAF medium dose (RAF-M, 50 mg/kg, i.g.), RAF low dose (RAF-L, 25 mg/kg, i.g.) groups. Fosinopril was used as the positive control. The drugs were dissolved in 0.5% CMC-Na and were orally administered once a day for 7 days. The control groups were only given solvent.

### Echocardiography

The mice were administered drugs or solvent for 7 days and then isoflurane (Ward Life Science and Technology Co., Ltd., Shenzhen, China) was used to anesthetize for 2D M-mode and B-mode echocardiography ultrasonic. The levels of left ventricular end-diastolic diameter (LVEDd), ejection fraction (EF), left ventricular end-systolic diameter (LVEDs) and fractional shortening (FS) were calculated for measurement of the cardiac function.

### Sample collection

After the echocardiography, 0.5% pentobarbital sodium were used to anaesthetize the mice at 50 mg/kg by intraperitoneal injection, and then they were sacrificed for collecting blood samples and heart samples. The serum and plasma were obtained through centrifugation for 20 min at 3000 rpm and then stored at –80 °C. The serum levels of lactate dehydrogenase (LDH) and creatine kinase-MB (CK-MB) were detected by Hitachi 17080 Automatic Biochemical Analyzer (Hitachi Co., Ltd., Japan). The plasma level of angiotensin II (AngII) was detected by ELISA. Heart was divided into two pieces along the ligature line and then the lower half was immediately put in liquid nitrogen for frozen, then it was stored at – 80 °C until testing. The left of the heart was prepared for paraffin section, which was placed in 4% paraformaldehyde for 72 h.

### Histological examination

After 72 h fixing, cardiac tissues were cut into 4 μm thin slices and prepared for pathological examination. The paraffin sections were stained with haematoxylin and eosin (HE), masson stain and sirius red stain, and then were observed with microscope.

### TUNEL assay

The cardiac tissue samples were embedded in paraffin and sectioned into 3 μm thick flakes to determine apoptotic cells. Paraffin sections were immersed two times with xylene for 5 min each and then putted in 100%, 95%, 90%, 80%, 70% ethanol solutions for 3 min, respectively. Then treated tissue with Proteinase K working solution for 15–30 min, at 21–37 °C. After incubation with 2'-deoxyuridine 5'-triphosphate for 1 h at 37 °C, the nucleus was counterstained with 4',6-Diamidino-2-phenylindole dihydrochloride (DAPI).

### qRT-PCR

The TRIzol reagent (Gibco-BRL, Paisley, UK) was used to extract total RNA according to the manufacturer's protocols. The concentration of the samples was determined by Nano Drop 2000 (Thermo Scientific, USA). Subsequently, the PCR Mix kit (Transgen Biotech Co., Beijing, China) was used to amplify the mice tissue cDNA. Afterwards, the expression of mRNA was measured by qRT-PCR: 1 cycle at 94 °C for 30 s and 40 cycles at 94 °C for 5 s and 50 °C for 30 s and then determined by melting curve. The primer sequences were showed as follows: p38 mitogen-activated protein kinases (p38 MAPK) F, CCTATCCTGGAAGAGCCATACT; p38 MAPK R, ACTTTGTCACGCTGACCAGAT; Bax F, AGACAGGGGCCTTTTTGCTAC, Bax R, AATTCGCCGGAGACACTCG; Bcl-2 F, GAGCCTGTGAGAGACGTGG, Bcl-2 R, CGAGTCTGTGTATAGCAATCCCA; GAPDH F, TATGACTCTACCCACGGCAAG, GAPDH R, TACTCAGCACCAGCATCACC.

### Immunofluorescence detection of tissue

After deparaffinized and immersed in xylene, the paraffin slices were eluted with an alcohol gradient. The tissue slices were placed in EDTA (pH 8.0) for antigen retrieval. The interval of power-off after 8 min at medium heat to boiling is 16 min and then low heat for 7 min to boiling. The sections were incubated with anti-p38 MAPK (8690 T, Cell Signaling Technology), anti-Bcl-2 (26593, Proteintech) and anti-Bax (50599, Proteintech) at 4 °C overnight. And then incubated with goat anti-rabbit IgG (LP1001B, ABGENT) at room temperature for 50 min. Counter-stain the cell nucleus with DAPI and incubate for 10 min in the dark at room temperature. After washing with PBS, the images were captured with the laser scanning confocal microscope (FV1000 Olympus, Japan).

### Cell viability assay

H9c2 cells (China Infrastructure of Cell Line Resources, Beijing, China) was cultured under the condition of 37 °C with 5% CO_2_ and the medium was DMEM (Corning, Manassas, VA, USA) mixed with 10% FBS (Corning, Manassas, VA, USA). DMSO (Sigma, USA) was used to dissolve RAF and the initial concentration was 40 mg/mL. Firstly, the H9c2 cells were seeded in 96-well plates with the density of 8 × 10^3^ cells/well for 24 h before incubating with RAF (1.25, 2.5, 5, 10, 20, 40, 80, 160, and 320 μg/mL) for 24 h. Secondly, we put the 96-well plates into the hypoxia box and added RAF at the concentrations of 0.63, 1.25, 2.5, 5, and 10 μg/mL for 8 h. Control group was cultured without the hypoxia box and other conditions were  the same. Finally, each well was added 100 μL CCK-8 and incubated for 2 h, then a microplate reader was used to measure the OD value of the plates at 450 nm.

### Immunofluorescence detection of cells

The H9c2 cells were seeded into 6-well plates and the density was 2 × 10^5^ cells/mL under the condition of 37 °C with 5% CO_2_ for 24 h. After removed the supernatant, each well was added 500 μL of PBS and discarded. Then the cells were fixed in 4% paraformaldehyde and were stained by Hoechst 33258 (1 μg/mL) for 10 min in dark. Afterwards, the cells were washed with PBS and dried while protected from light. The fluorescence microscope (Leica Micro systems GmbH) was used to observe the apoptotic cells at 350 nm and 460 nm.

### Western blot

Myocardial tissue (50 mg) and the H9c2 cells treated with hypoxia were harvested and fragmented in RIPA lysis buffer (Applygen Technologies Inc., Beijing, China) supplemented with 1% protease inhibitor and phosphatase inhibitor (PierceTM, Thermo Fisher Scientific, USA). After the sample content was measured by BCA method (Applygen Technologies Inc., Beijing, China), the protein was boiled at 99 °C for 10 min. The protein samples of tissue and cells were separated by 10% SDS-PAGE gels and then they were transferred onto PVDF membranes (Millipore, Boston, MA, USA). After that, the primary antibodies (1:1000 or 1:2000, please refer to the Additional file 1: Table S1) and secondary antibodies (1:2000) were incubated with the membranes at 4 °C overnight and at room temperature for 2 h, respectively. Then the bands were reacted with ECL (ECL Plus, GE Healthcare, USA) in the dark for 1 min, and were analyzed through the Image lab software.

### Statistical analysis

In the study, all data were showed as the mean ± SEM and analyzed by GraphPad Prism 5.0 (GraphPad Software, Inc., San Diego, CA, USA). The differences among groups were calculated by one-way analysis of variance (ANOVA) with post hoc Dunnett’s test. *P* < 0.05 represented significant.

## Results

### LCMS-IT-TOF profiles of RAF and PAF

HPLC chromatograms showed that RAF contains ingredients during 0–40 min retention time with moderate and high polarity, whereas PAF mainly consists of hydrophilic constituents in the first 20 min (please refer to the Additional file 1: Fig. S1). Based on data analysis of LC–MS and those previously isolated compounds [13], 14 major peaks of alkaloids were assigned as *N*-trans-p-coumaroy lnoradrenline (**1**); N-trans-p-coumaroyloctopamine (**2**); magnoflorine (**3**); N-trans-feruloyloctopamine (**4**); berberine (**5**); dehydrocheilanthifoline (**6**); isomer-dehydrocheilanthifoline (**7**); tetrahydropalmatine (**8**); bicuculine (**9**); 6,7-methylenedioxy-2-(6-acetyl-2,3- methylenedioxybenzyl)-1(2H)-isoquinolinone (**10**); protopine (**11**); allocryptopine (**12**); hendersine B (**13**); stylopine (**14**).

### Effect on echocardiography

**Fig. 1:**
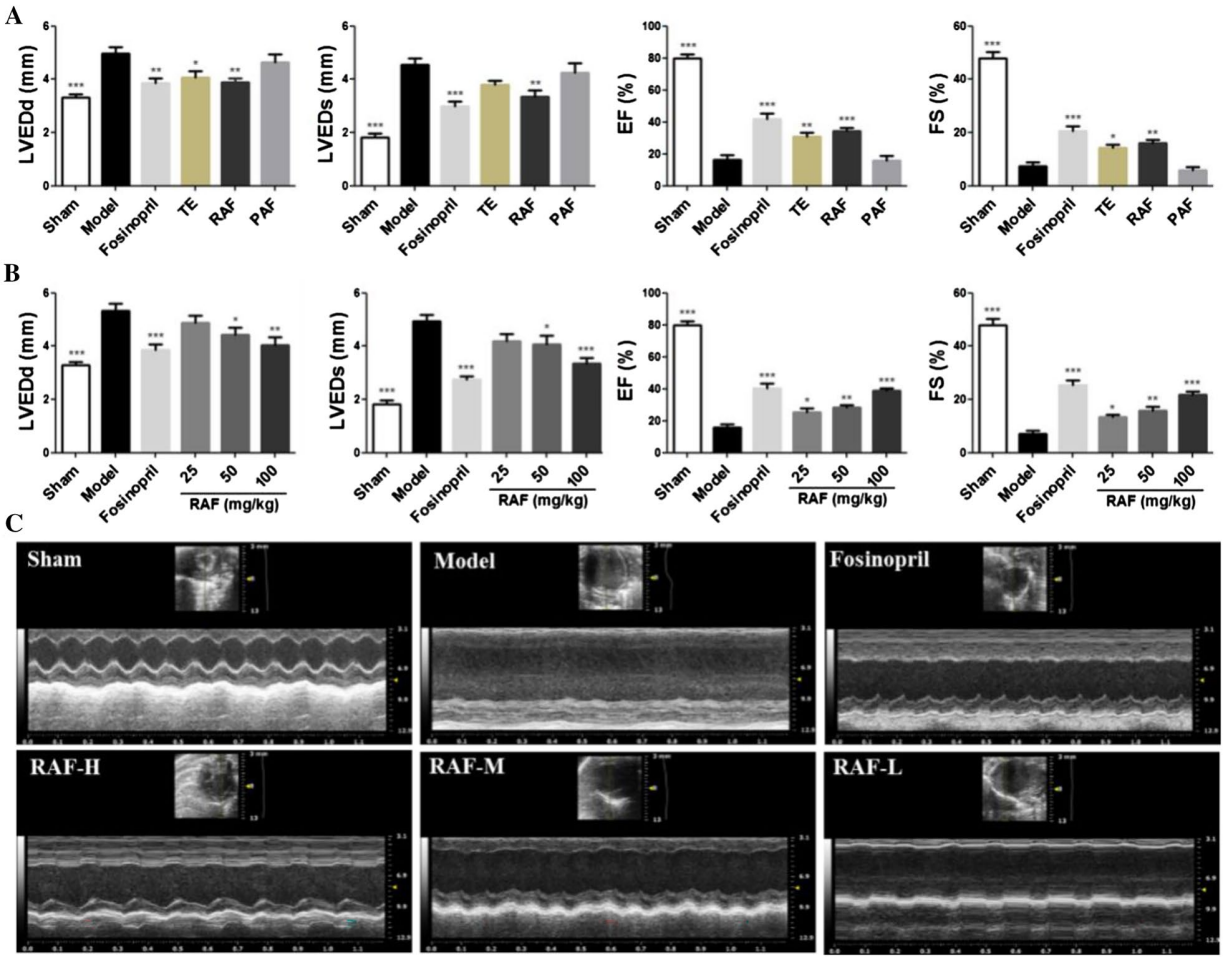
Effect on cardiac function in AMI mice. **A** Mice were administered with TE (400 mg/kg, i.g.), RAF (100 mg/kg, i.g.), PAF (300 mg/kg, i.g.), 0.5% CMC-Na (i.g.) or fosinopril (10 mg/kg, i.g.) for 7 days. **B** Mice were administered with RAF-L (25 mg/kg, i.g.), RAF-M (50 mg/kg, i.g.), RAF-H (100 mg/kg, i.g.), 0.5% CMC-Na (i.g.) or fosinopril (10 mg/kg, i.g.) for 7 days. The LVEDd, LVEDs, EF and FS of AMI mice were measured by echocardiography. Data are represented as the mean ± SEM of 8 mice per group. ^*^P < 0.05, ^**^P < 0.01, and ^***^P < 0.001 compared with the Model group. **C** The representative echocardiography images

Compared with the sham group, the values of LVEDd and LVEDs were increased significantly and the values of EF and FS were decreased significantly (*P* < 0.001, *P* < 0.001) in the model group. The TE and RAF group could decrease the levels of LVEDd and LVEDs and increase the levels of EF and FS, as shown in Fig. 1A. Moreover, after treatment for seven days, RAF existed the same effect compared with TE in ameliorating mice cardiac function, but PAF showed negative result, as shown in Fig. 1B, 1C. As descripted above, it suggested that RAF contributes to the cardioprotective effect whereas PAF didn’t.

### Effect on the levels of CK-MB, LDH and AngII

**Fig. 2:**
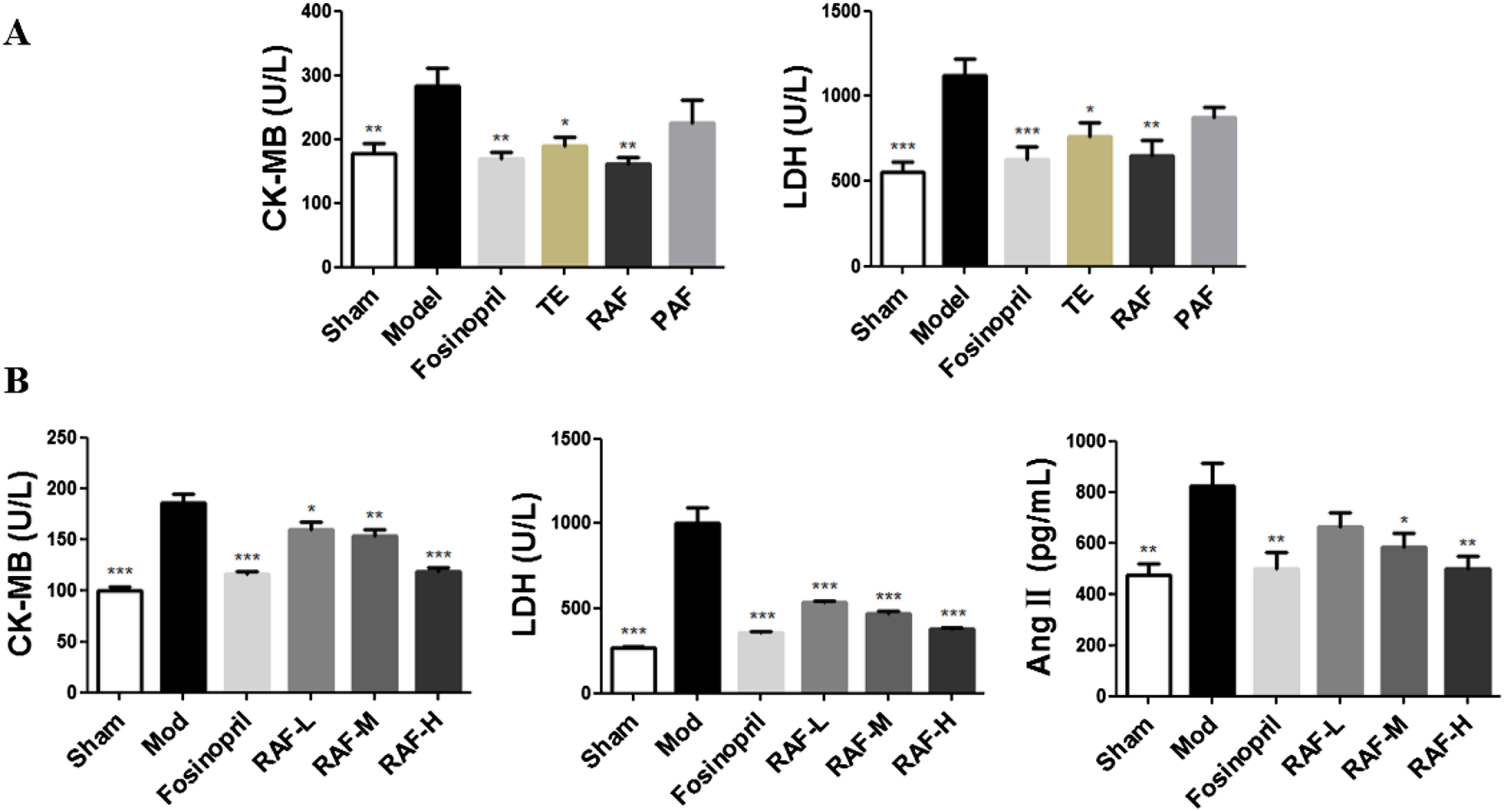
Effect on CK-MB, LDH and AngII in AMI mice. **A** Mice were administered with TE (400 mg/kg, i.g.), RAF (100 mg/kg, i.g.), PAF (300 mg/kg, i.g.), 0.5% CMC-Na (i.g.) or fosinopril (10 mg/kg, i.g.) for 7 days. The serum levels of CK-MB, LDH were analyzed by Automatic Biochemical Analyzer. **B** Mice were administered with RAF-L (25 mg/kg, i.g.), RAF-M (50 mg/kg, i.g.), RAF-H (100 mg/kg, i.g.), 0.5% CMC-Na (i.g.) or fosinopril (10 mg/kg, i.g.) for 7 days. The serum levels of CK-MB, LDH were analyzed by Automatic Biochemical Analyzer and the plasma level of AngII was detected by ELISA. Data are represented as the mean ± SEM of 8 mice per group. ^*^P < 0.05, ^**^P < 0.01, and ^***^P < 0.001 compared with the Model group

The levels of LDH, CK-MB, and AngII were markedly high (*P* < 0.001) in the model group. Compared with the model group, RAF and TE could reduce the rising levels of LDH and CK-MB in serum (*P* < 0.01, *P* < 0.05), whereas PAF had no significant influence (Fig. 2A). Our further research found that RAF also improved the myocardial enzymes and mitigated the increase in plasma AngII level (Fig. 2B).

### Effect on myocardial tissue pathology

**Fig. 3:**
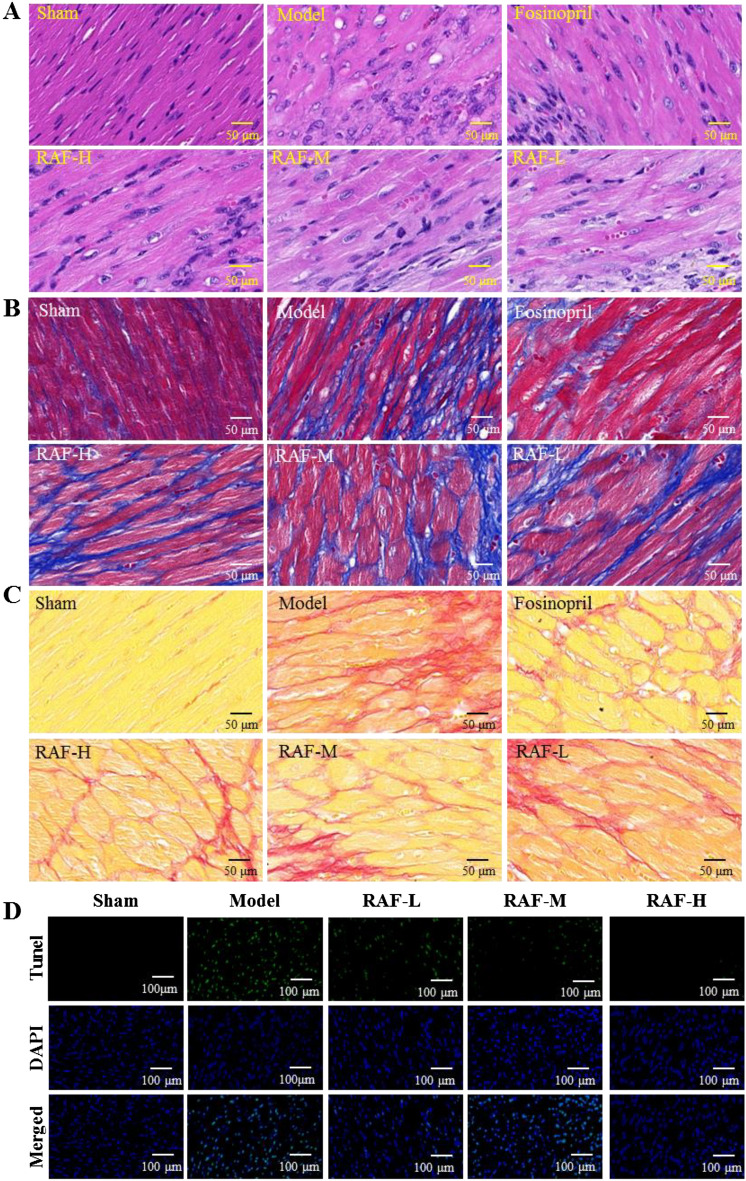
Effect on myocardial histopathology and apoptosis after AMI modeling. Mice were administered with RAF-L (25 mg/kg, i.g.), RAF-M (50 mg/kg, i.g.), RAF-H (100 mg/kg, i.g.), 0.5% CMC-Na (i.g.) or fosinopril (10 mg/kg, i.g.) for 7 days. **A** HE staining (images magnification at 400 ×); **B** Masson staining (collagen is stained blue, myocardial cells are stained red) (images magnification at 400 ×); **C** Sirius red staining (collagen is stained red, myocardial cells are stained yellow) (images magnification at 400 ×); **D** TUNEL staining (images magnification at 40 ×). n = 3

HE staining displayed that the cells were normal and arranged regularly in the sham group, while a great quantity of inflammatory cells infiltrate in the interstitium related to the model group (Fig. 3A). MASSON and Sirius red staining in the sham group showed that the interstitium contained a small amount of collagen fibers and no fibroblast proliferation, but the destruction of the collagen network and the myocardial fibrosis were serious after modeling. However, treatment RAF for 7 days, the inflammatory cell infiltration and collagen deposition were decreased, demonstrating the ischemic injury of mice myocardium were improved (Fig. 3B, C).

The TUNEL assay result illustrated that the myocardial cells after modeling were more apoptotic since the blue-green fluorescence was increased. RAF treatment markedly reduced myocardial apoptosis, protecting the ischemic myocardial damage (Fig. 3D).

### Effect on p38 MAPK, Bax, Bcl-2 mRNA expression

**Fig. 4:**
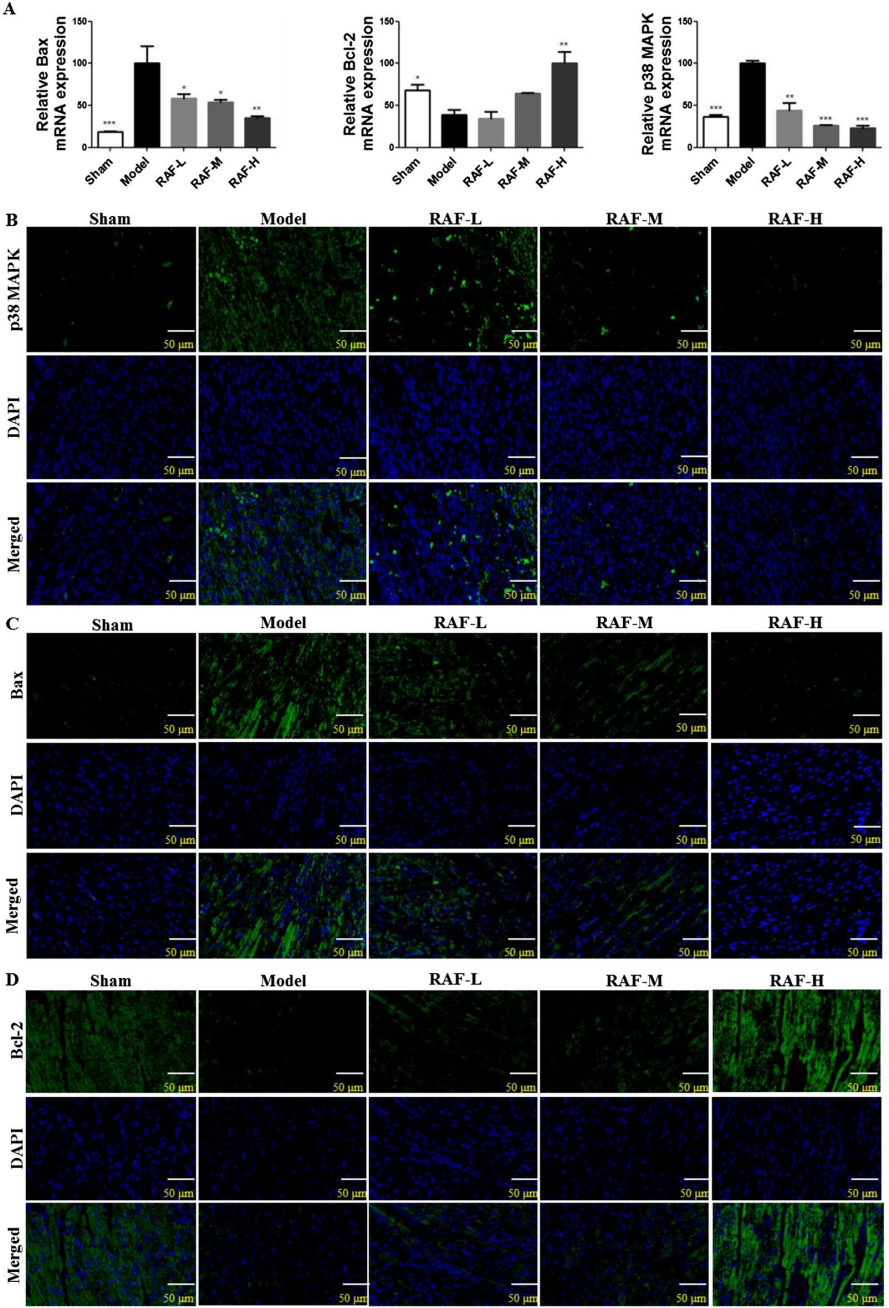
Effect on p38 MAPK, Bax and Bcl-2 in heart tissue. Mice were administered with RAF-L (25 mg/kg, i.g.), RAF-M (50 mg/kg, i.g.), RAF-H (100 mg/kg, i.g.) or 0.5% CMC-Na (i.g.) for 7 days. The expressions of p38 MAPK, Bax and Bcl-2 were analyzed by qRT-PCR (**A**) and immunofluorescence staining (**B**–**D**). Data are represented as the mean ± SEM of 3 mice per group. ^*^P < 0.05, ^**^P < 0.01, and ^***^P < 0.001 compared with the Model group

The expression of p38 MAPK, Bcl-2 and Bax mRNA were detected through qRT-PCR. In model group, the mRNA expression of p38 MAPK and Bax was significantly up-regulated, suggesting that myocardial ischemia could increase p38 MAPK expression. RAF down-regulated the mRNA levels of p38 MAPK and Bax, and up-regulated the mRNA level of the anti-apoptotic gene Bcl-2 (Fig. 4A).

### Effect on p38 MAPK, Bax, Bcl-2 immunofluorescence staining

The green fluorescence intensity was increased in the model group, indicating that the content of p38 MAPK and Bax protein increased. Those signals of the treatment group were weaker than the model group, suggesting that RAF may inhibit the protein expression of p38 MAPK and Bax (Fig. 4B, C). While the signals of Bcl-2 immunofluorescence were weak in the model group, after administered with RAF, the signals were enhanced (Fig. 4D).

### Effects on p38 MAPK-mediated apoptotic proteins

**Fig. 5:**
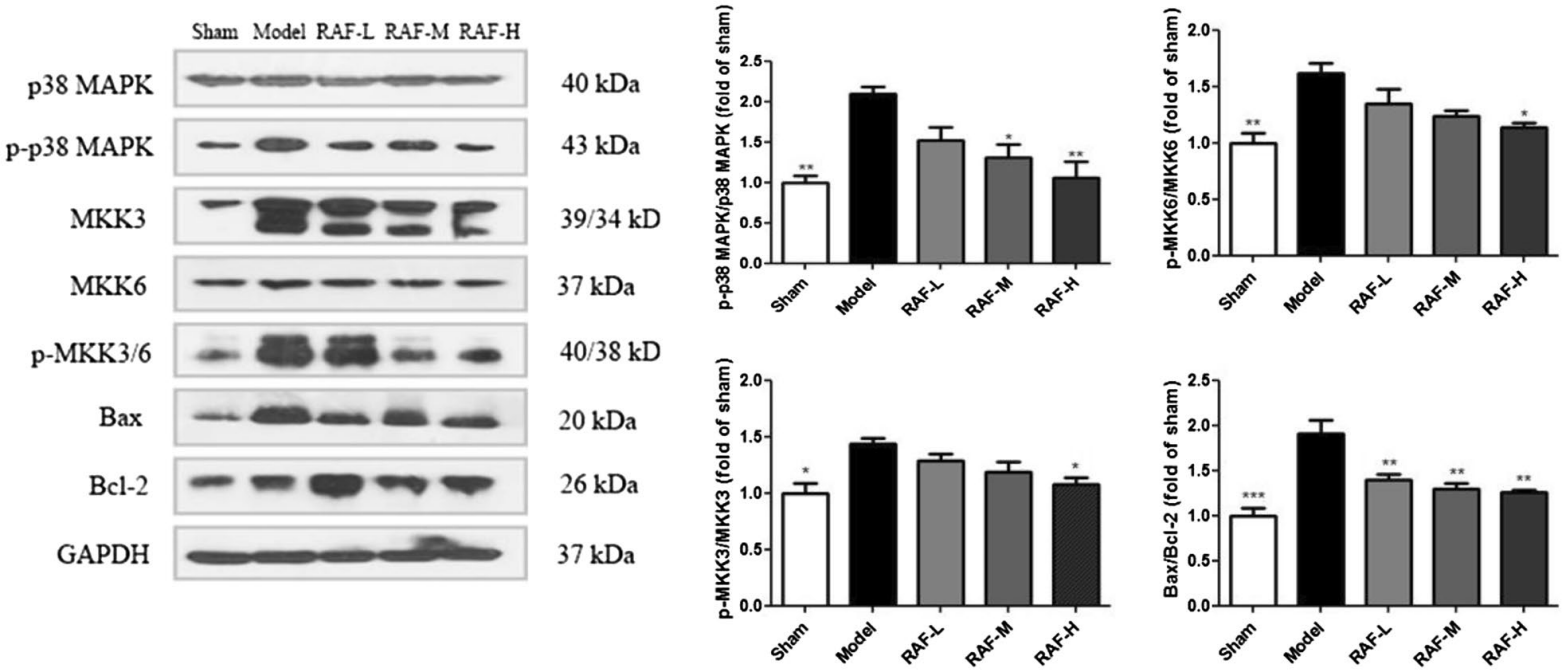
Effect on protein expression of p-p38 MAPK, p-MKK3/6 and Bax/Bcl-2 in AMI mice. Mice were administered with RAF-L (25 mg/kg, i.g.), RAF-M (50 mg/kg, i.g.), RAF-H (100 mg/kg, i.g.) or 0.5% CMC-Na (i.g.) for 7 days. The protein expression was detected by western blot. Data are represented as the mean ± SEM of 3 mice per group. ^*^P < 0.05, ^**^P < 0.01, and ^***^P < 0.001 compared with the Model group

Compared with the sham group, the expression of p-p38 MAPK, p-MKK3/6 and Bax proteins were obviously increased in mice after myocardial infarction, while RAF had a significant inhibitory effect against apoptosis by down-regulating the expression of these proteins. The RAF administration group also up-regulated the anti-apoptotic protein expression of Bcl-2 to varying degrees. The protein results revealed that the anti-AMI effect of RAF was related to the p38 MAPK signaling pathway (Fig. 5).

### Effects on H9c2 cells viability

**Fig. 6:**
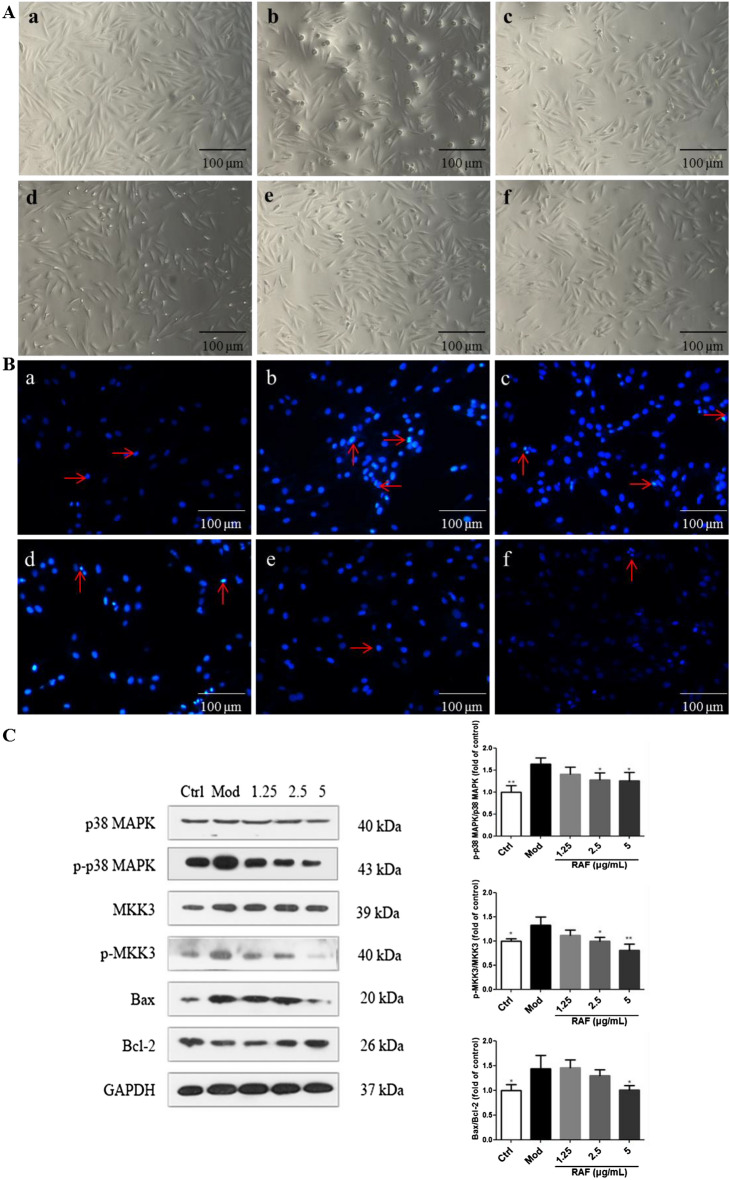
Effect on the viability and protein expression of H9c2 cells in hypoxia test. **a** Control group; **b** Model group; **c** RAF group (0.63 μg/mL); **d** RAF group (1.25 μg/mL); **e** RAF group (2.5 μg/mL); **f** RFA group (5 μg/mL). After treatment with RAF or vehicle control for 8 h, the viability of H9c2 cells was examined by **A** Morphological observations (images magnification at 10 ×) and **B** Hoechst 33258 fluorescence detection (images magnification at 40 ×). **C** After treatment with RAF (1.25, 2.5, and 5 μg/mL) or vehicle control for 8 h, the protein expression of p-p38 MAPK, p-MKK3 and Bax/Bcl-2 was analyzed by western blot. ^*^P < 0.05, ^**^P < 0.01 compared with the Mod group

After 24 h of administration, cell survival rates of RAF (1.25–10 μg/mL) groups were not lower than the control group (*P* > 0.05), indicating that RAF had no apparently cytotoxic to H9c2 cells at the concentration of lower than 10 μg/mL. After 8 h of culture under hypoxia, serum-free and low glucose conditions, RAF significantly improved cell viability at the concentration of 1.25, 2.5, and 5 μg/mL, indicating that RAF had protective effect against hypoxia-induced injury (Fig. 6A and the Additional file 1: Fig. S3).

### Effects on apoptosis of H9c2 cells

After treatment of RAF (1.25, 2.5, and 5 μg/mL), the fluorescence intensity was remarkably decreased, indicating that RAF could suppress apoptosis. Through the microscope we found that the living cells were increased and the dead cells were decreased in the RAF-treated group, further indicating that RAF could improve the survival rate of cells (Fig. 6B).

### Effects on protein expression of H9c2 cells

When myocardial ischemia occurred, the expression of p-p38 MAPK, p-MKK3 and Bax were up-regulated, which suggested that hypoxia injury could activated the p38 MAPK-mediated apoptotic signaling pathway. However, RAF reversed the tendency of p-p38 MAPK, p-MKK3 and Bax/Bcl-2 expression compared to the model group (Fig. 6C). The above results showed that RAF exhibited anti-apoptotic effect via the p38 MAPK signaling pathway.

### Discussion

The present results showed that TE (400 mg/kg) and RAF (100 mg/kg) treatments could ameliorate mice cardiac function, and RAF (25–100 mg/kg) reduced the increased expression of p38 MAPK, MKK3, and MKK6 both in mice and H9c2 cells, indicating that RAF exerts the cardioprotective effect against ischemic injury through inhibiting apoptosis underlying p38 MAPK signaling pathway.

Coronary artery ligation leads to interruption of blood supply. After MI, oxygen supply decreases and energy metabolism are abnormal, and the heart fails to maintain normal work [14]. Continuous ischemia causes a wide range of myocardial infarction, further amplifies the inflammatory response, and aggravates myocardial damage. Apoptosis is one of the main ways of myocardial injury caused by coronary artery occlusion, and there are many apoptotic cells in ischemic myocardial injury [15]. More and more researches gradually verified that apoptosis is one of the most importantly mode of cardiomyocyte death when AMI happened [16–18]. Therefore, inhibiting myocardial cell apoptosis after myocardial infarction is one of the important treatment measures to improve cardiac function [19, 20]. As a key factor of myocardial apoptosis signal transduction, p38 MAPK is involved in a series of processes after AMI [21, 22]. Ischaemic injury may lead to mitochondrial dysfunction, and p38 MAPK signal pathway mainly causes cell apoptosis by affecting cell mitochondrial pathway. The upstream activators of p38 MAPK are MKK3, and MKK6. Under stress, MKK3 and MKK6 are activated, and then p38 MAPK is activated to activate apoptosis. In addition, the Bcl-2 family of proteins is also related to the regulation of ischaemia-induced apoptosis. The up-regulation of Bax apoptotic protein expression can lead to mitochondrial dysfunction and promote apoptosis [23–25]. Our findings depicted evident that RAF restored of MKK3, MKK6, and p38 phosphorylation and increased the ratio of Bcl-2 / Bax to attenuate cardiomyocyte apoptosis.

By analysis of LC–MS data, 14 alkaloids were characterized in RAF, including the major ones (peaks **3**, **6**–**12**) and minor in its contents (peaks **1**, **2**, **4**, **5**, **13**, and **14**). Among them, compounds **3, 6** and **11–14** were identified from TE in our previous study. Some alkaloids had the effects of anti-inflammatory and inhibiting platelet aggregation, which benefits to the anti-AMI activity of TE [26, 27]. Some alkaloids had also been reported in anti-oxidation and anti-apoptosis [28], which further explained the contribution of RAF to anti-AMI effect. At the same time, it suggested that TCM played an overall role with the multi-component and multi-pathway paradigm.

PAF accounting for 75% of TE in mass ratio didn’t show the effect against AMI and meanwhile, PAF had little signal in LC–MS. Whether PAF could contribute to other aspects of traditional efficacy and which substances could exert efficacy need follow-up study.

## Conclusion

In summary, both the rich-alkaloid fraction and total extract of *Corydalis hendersonii* Hemsl., one of Tibetan folk medicines, have the cardioprotective effect against AMI mice. The rich-alkaloid fraction limits myocardial ischemia through attenuating the cardiomyocyte apoptosis underlying the mechanism of p38 MAPK signaling pathway in H9c2 cells and mice. The major isoquinoline and phenylpropionamide alkaloids contribute to the efficacy and may be considered as the biologically active substances against ischemic heart disease. Our research provides the substantial foundation for the development of potential drugs for the treatment of ischemic heart diseases, and provides a reference for utilization of natural CH resource as the traditional medicines.

## Supplementary Information

**Additional file 1: Table S1.** Primary antibodies used in western blot experiment. **Table S2.** HR-ESI-MS data of compounds. **Fig. S1.** HPLC-DAD (A) and positive-mode (B) LCMS-IT-TOF chromatograms of RAF, and HPLC-DAD (C) and positive-mode (D) LCMS-IT-TOF chromatograms of PAF. Peaks 1: N-trans-p-coumaroy lnoradrenline, 2: N-trans-p-coumaroyloctopamine, 3: magnoflorine, 4: N-trans-feruloyloctopamine, 5: berberine, 6: dehydrocheilanthifo-line, 7: isomer-dehydrocheilanthifoline, 8: tetrahydropalmatine, 9: bicuculine, 10: 6,7-methylenedioxy-2-(6-acetyl-2,3-methylenedioxybenzyl)-1(2H)-isoquinolinone, 11: protopine, 12: allocryptopine, 13: hendersine B, 14: stylopine. **Fig. S2.** Chemical structures of 14 alkaloids in RAF of *Corydalis hendersonii* Hemsl. **Fig. S3.** (A) The cytotoxicity of RAF on H9c2 cells was detected by CCK-8 assay. Data are represented as the mean ± SEM of three independent experiments. **P < 0.01, ***P < 0.001, compared with the ctrl (control) group. (B) Detection of H9c2 cell viability after incubation in hypoxic serum-free medium for 8 h. Data are represented as the mean ± SEM of three independent experiments. *P < 0.05, **P < 0.01, ***P < 0.001, compared with the Model group.

## Data Availability

Data can be shared with the author's consent.

## Abbreviations

CH: *Corydalis hendersonii* Hemsl.
TE: Total extract
RAF: Rich alkaloids fraction
PAF: Poor alkaloidal fraction
HAPC: High-altitude polycythemia
AMI: Acute myocardial infarction
LVEDd: Left ventricular end-diastolic diameter
LDH: Lactate dehydrogenase
EF: Ejection fraction
LVEDs: Left ventricular end-systolic diameter
FS: Fractional shortening
AngII: Angiotensin II
CK-MB: Creatine kinase-MB
Bax: Bcl-2-associated X protein
IHD: Ischemic heart disease
BV/h: Bed volumes per hour
DAD: Diode array detector
MKK: MAP kinase kinase
LCMS-IT-TOF: Liquid chromatography mass spectrometry-ion trap-time of flight
p38 MAPK: P38 mitogen-activated protein kinases
