# Supplementary material for: The alkaloids of Corydalis hendersonii Hemsl. contribute to the cardioprotective effect against ischemic injury in mice by attenuating cardiomyocyte apoptosis via p38 MAPK signaling pathway
Source: Chin Med. 2023 Mar 17;18:29. doi: 10.1186/s13020-023-00726-8 (PMC10021936; doi:10.1186/s13020-023-00726-8)
Supplement: Supplementary file 1 — Additional file 1: Table S1. Primary antibodies used in western blot experiment. Table S2. HR-ESI-MS data of compounds. Fig. S1. HPLC-DAD (A) and positive-mode (B) LCMS-IT-TOF chromatograms of RAF, and HPLC-DAD (C) and positive-mode (D) LCMS-IT-TOF chromatograms of PAF. Peaks 1: N-trans-p-coumaroy lnoradrenline, 2: N-trans-p-coumaroyloctopamine, 3: magnoflorine, 4: N-trans-feruloyloctopamine, 5: berberine, 6: dehydrocheilanthifo-line, 7: isomer-dehydrocheilanthifoline, 8: tetrahydropalmatine, 9: bicuculine, 10: 6,7-methylenedioxy-2-(6-acetyl-2,3-methylenedioxybenzyl)-1(2H)-isoquinolinone, 11: protopine, 12: allocryptopine, 13: hendersine B, 14: stylopine. Fig. S2. Chemical structures of 14 alkaloids in RAF of Corydalis hendersonii Hemsl. Fig. S3. (A) The cytotoxicity of RAF on H9c2 cells was detected by CCK-8 assay. Data are represented as the mean ± SEM of three independent experiments. **P < 0.01, ***P < 0.001, compared with the ctrl (control) group. (B) Detection of H9c2 cell viability after incubation in hypoxic serum-free medium for 8 h. Data are represented as the mean ± SEM of three independent experiments. *P < 0.05, **P < 0.01, ***P < 0.001, compared with the Model group. [file 13020_2023_726_MOESM1_ESM.docx]

*Supporting information for*

**The alkaloids of *Corydalis hendersonii* Hemsl. contribute to the cardioprotective effect against ischemic injury in mice by attenuating cardiomyocyte apoptosis via p38 MAPK signaling pathway**

Fuxing Ge^1,†^, Xiaoli Gao^1,†^, Xiaochun Zhou^1^, Junjun Li^1^, Xiaojing Ma^1^, Meiwen Huang^1^, Sana Wuken^1^, Pengfei Tu^1^, Chao An^2*^ and Xingyun Chai^1*^

Affiliation:

^1^ Modern Research Center for Traditional Chinese Medicine, Beijing Research Institute of Chinese Medicine, Beijing University of Chinese Medicine, Beijing 102488, P. R. China

^2^ Dongfang Hospital, Beijing University of Chinese Medicine, Beijing 100078, P. R. China

Corresponding authors:

Xingyun Chai (Tel/Fax: 8610 6428 6350, E-mail: [xingyunchai@yeah.net](mailto:xingyunchai@yeah.net))

Chao An (Tel/Fax: 8610 6428 6458, E-mail: [annie_bucm@126.com](mailto:annie_bucm@126.com))

^†^ The first two authors contributed to the paper equally.

**Table S1** Primary antibodies used in western blot experiment

| **Protein** | **Primary antibody** | **Concentration** |
| --- | --- | --- |
| p38 MAPK | Anti-p38 MAPK 8690T CST | 1:1000 |
| p-p38 MAPK | Anti-p-p38 MAPK 4511T CST | 1:1000 |
| MKK3 | Anti-MKK3 ab195037 Abcam | 1:1000 |
| MKK6 | Anti-MKK6 ab33866 Abcam | 1:1000 |
| p-MKK3/6 | Anit-MKK3/6 12280 CST | 1:1000 |
| Bax | Anti-Bax 50599 Proteintech | 1:2000 |
| Bcl-2 | Anti-Bcl2 26593 Proteintech | 1:2000 |
| GAPDH | GAPDH 51332S CST | 1:1000 |


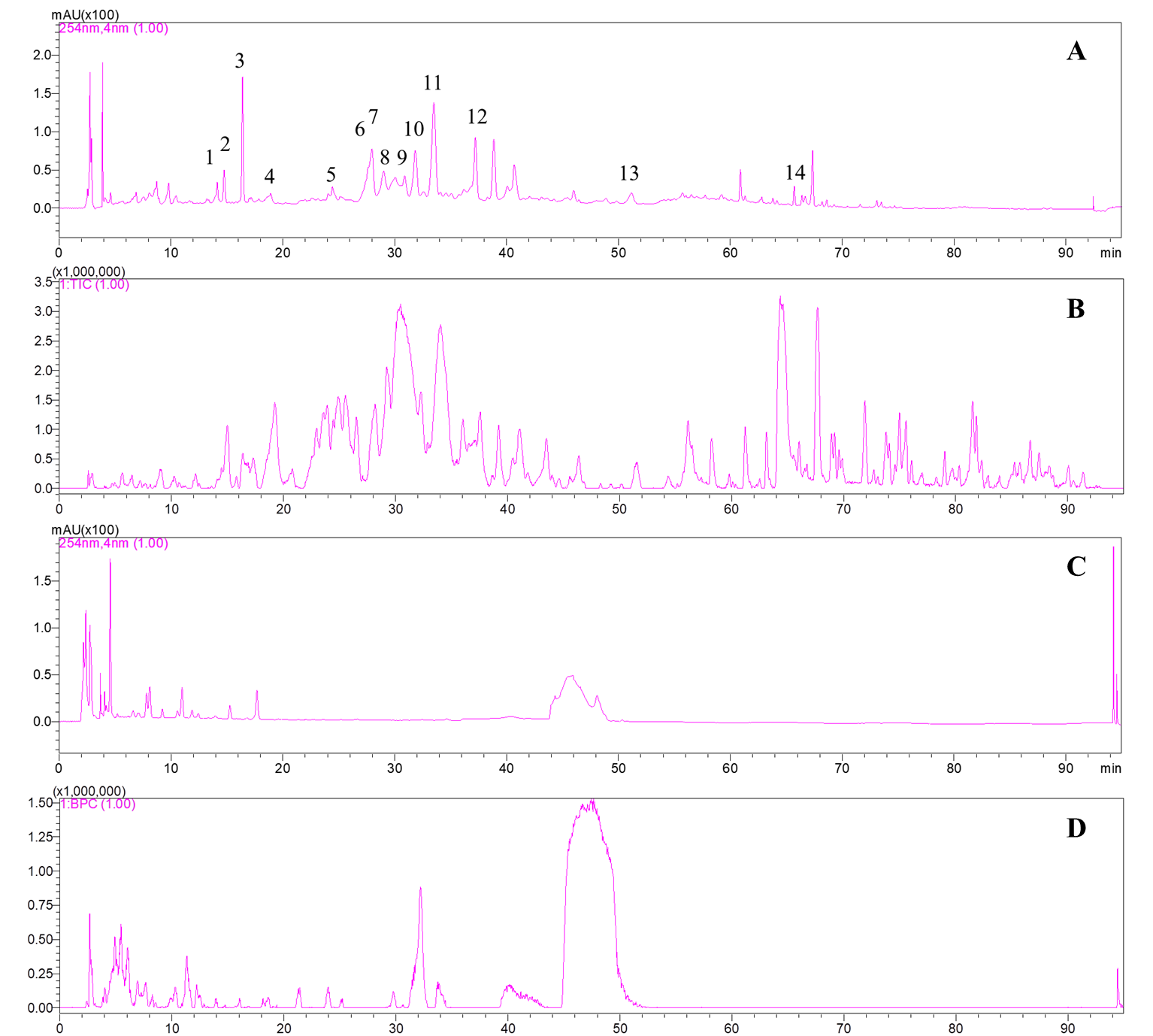


**Fig. S1** HPLC-DAD (**A**) and positive-mode (**B**) LCMS-IT-TOF chromatograms of RAF, and HPLC-DAD (**C**) and positive-mode (**D**) LCMS-IT-TOF chromatograms of PAF. Peaks **1**: *N*-trans-p-coumaroy lnoradrenline, **2**: *N*-trans-*p*-coumaroyloctopamine, **3**: magnoflorine, **4**: *N*-trans-feruloyloctopamine, **5**: berberine, **6**: dehydrocheilanthifo-

line, **7**: isomer-dehydrocheilanthifoline, **8**: tetrahydropalmatine, **9**: bicuculine, **10**: 6,7-methylenedioxy-2-(6-acetyl-2,3-methylenedioxybenzyl)-1(2H)-isoquinolinone, **11**: protopine, **12**: allocryptopine, **13**: hendersine B, **14**: stylopine.

**Fig. S2** Chemical structures of 14 alkaloids in RAF of *Corydalis hendersonii* Hemsl.

**Table S2** HR-ESI-MS data of compounds

| No. | Structure | *m/z* |
| --- | --- | --- |
| 1 |  | 316.1524 [M + H]^+^ (C_17_H_18_NO_5_) |
| 2 |  | 300.1642 [M + H]^+^ (C_17_H_18_NO_4_) |
| 3 |  | 342.1694 [M]^+^ (C_20_H_24_NO_4_) |
| 4 |  | 330.1939 [M + H]^+^ (C_18_H_20_NO_5_) |
| 5 |  | 336.1654 [M]^+^ (C_20_H_18_NO_4_) |
| 6 |  | 322.1089 [M]^+^ (C_19_H_16_NO_4_) |
| 7 |  | 322.1081 [M]^+^ (C_19_H_16_NO_4_) |
| 8 |  | 356.1492 [M+H]^+^ (C_21_H_26_NO_4_) |
| 9 |  | 406.1613 [M+K]^+^ (C_20_H_17_NO_6_K) |
| 10 |  | 366.0964 [M + H]^+^ (C_20_H_16_NO_6_) |
| 11 |  | 354.1686 [M + H]^+^ (C_20_H_20_NO_5_) |
| 12 |  | 370.1639 [M + H]^+^ (C_21_H_24_NO_5_) |
| 13 |  | 366.0598 [M + H]^+^ (C_19_H_12_NO_7_) |
| 14 |  | 324.1412 [M + H]^+^ (C_19_H_18_NO_4_) |

The NMR data of the 14 compounds list below:

(**1**) ^1^H NMR (CD_3_OD, 500 MHz): *δ*_H_ 7.45 (1H, d, *J* = 15.5 Hz, H-7), 6.52 (1H, d, *J* = 16.0 Hz, H-8), 3.51 and 3.38 (2H, m, *J* = 13.0 Hz，5.5 Hz, H-8′), 4.28 (1H, m, *J* = 8.0 Hz, H-7′), 6.86 (1H, d, *J* = 1.5 Hz, H-2′), 6.78 (1H, d, *J* = 1.5 Hz, H-5′), 6.83 (1H, d, *J* = 8.5 Hz, 1.5Hz, H-6′), 7.40 (2H, d, *J* = 8.0Hz, H-2, 6). ^13^C NMR (CD_3_OD, 125 MHz): *δ*_C_ 169.4 (C-9), 159.4 (C-4), 150.0 (C-3′), 147.1 (C-4′), 142.3 (C-7), 134.6 (C-1′), 130.2 (C-2, 6), 128.1 (C-1), 119.2 (C-6′), 118.5 (C-8), 116.4 (C-3, C-5), 116.1 (C-5), 115.9 (C-5′), 115.0 (C-2′), 73.4 (C-7′), 48.2 (C-8′).

(**2**) ^1^H NMR (CD_3_OD, 500 MHz): *δ*_H_ 7.45 (1H, d, *J* = 15.5 Hz, H-7), 6.52 (1H, d, *J* = 16.0 Hz, H-8), 3.51 and 3.38 (2H, m, *J* = 13.0 Hz, 5.5 Hz, H-8′), 4.28 (1H, m, *J* = 8.0 Hz, H-7′), 7.18 (2H, d, *J* = 8.0 Hz, H-2′, 6′)，6.75 (2H, d, *J* = 8.0 Hz, H-3′, 5′), 6.79 (1H, d, *J* = 8.5 Hz, H-3, 5), 7.40 (2H, d, *J* = 8.5Hz, H-2, 6). ^13^C NMR (CD_3_OD, 125 MHz): *δ*_C_ 169.4 (C-9), 159.4 (C-4), 116.0 (C-3′), 147.1 (C-4′), 142.3 (C-7), 134.6 (C-1′), 130.2 (C-2, 6), 128.1 (C-1), 119.2 (C-6′), 118.5 (C-8), 116.4 (C-3, C-5), 116.1 (C-5), 115.9 (C-5′), 115.0 (C-2′), 73.4 (C-7′), 48.2 (C-8′).

(**3**) ^1^H NMR (DMSO-*d*_6_, 500 MHz): *δ*_H_ 6.36 (1H, d, *J* = 8.0 Hz, H-8), 6.60 (1H, d, *J* = 8.0 Hz, H-9), 6.51 (1H, s, H-3), 4.33 (1H, d, *J* = 13.0 Hz, 8.0 Hz, H-6a), 3.56 (2H, m, H-5), 3.67 (3H, s, OCH_3_), 3.69 (3H, s, OCH_3_), 3.30 (3H, s, *N-*CH_3_*-α*), 2.87 (3H, s, *N-*CH_3_*-β*), 3.14 (1H, m, *J* = 28.0 Hz, H-7*α*), 2.59 (1H, m, *J* = 8.0 Hz, H-7*β*), 2.73 (1H, m, *J* = 17.5 Hz, H-4*α*), 3.12 (1H, m, *J* = 17.5 Hz, H-4*β*)。^13^C NMR (DMSO-*d*_6_, 125 MHz): *δ*_C_ 152.2 (C-11), 151.8 (C-1), 151.5 (C-2), 150.2 (C-10), 125.1 (C-7a), 123.0 (C-11a), 120.0 (C-1b), 111.8 (C-3a), 109.7 (C-9), 108.6 (C-3), 69.1 (C-6a), 60.4 (C-5), 55.6 (OCH_3_), 55.2 (OCH_3_), 52.6 (*N-*CH_3_*-α*), 42.4 (*N-*CH_3_*-β*), 30.4 (C-7), 23.2 (C-4).

(**4**) ^1^H NMR (CD_3_OD, 500 MHz): *δ*_H_ 7.44 (1H, d, *J* = 15.5 Hz, H-7), 7.24 (2H, d, *J* = 8.5 Hz, H-2′, H-6′), 6.99 (1H, d, *J* = 8.0 Hz, 1.5 Hz, H-2), 6.48 (1H, d, *J* = 15.5 Hz, H-8), 6.78 (2H, d, *J* = 8.5 Hz, H-3′, H-5′), 6.78 (1H, d, *J* = 8.0 Hz, H-5), 7.08 (1H, d, *J* = 1.5Hz, H-2), 4.75 (1H, dd, *J* = 5.0Hz, H-7′), 3.83 (3H, s, OCH_3_), 3.47 (1H, m, H-8′*α*), 3.55 (1H, m, H-8′*β*). ^13^C NMR (CD_3_OD, 125 MHz): *δ*_C_ 169.4 (C-9), 157.9 (C-4′), 149.7 (C-4), 149.1 (C-3), 142.3 (C-7), 134.6 (C-1′), 128.4 (C-2′, 6′), 128.1 (C-1), 123.2 (C-6), 118.5 (C-8), 116.4 (C-3′, C-5′), 116.1 (C-5), 111.5 (C-2), 73.4 (C-7′), 56.3 (3-OCH_3_), 48.2 (C-8′).

(**5**) ^1^H NMR (CD_3_OD, 500 MHz): *δ*_H_ 9.75 (1H, s, H-8), 8.67 (1H, s, H-13), 8.11 (1H, d, *J* = 9.0 Hz, H-11), 7.98 (1H, d, *J* = 9.0 Hz, H-12), 7.63 (1H, s, H-1), 6.95 (1H, s, H-4), 6.10 (2H, s, OCH_2_O), 4.93 (2H, t, *J* = 13.0, 6.5 Hz, CH_2_), 3.26 (2H, t, *J* = 13.0, 6.5 Hz, CH_2_), 4.21 (3H, s, OCH_3_), 4.13 (3H, s, OCH_3_). ^13^C NMR (CD_3_OD, 125 MHz): *δ*_C_ 152.2 (C-2), 152.0 (C-9), 149.9 (C-3), 146.4 (C-10), 145.8 (C-8), 139.7 (C-14), 135.1 (C-4a), 131.9 (C-1a), 128.1 (C-11), 124.5 (C-12), 123.3 (C-8a), 121.8 (C-13), 111.4 (C-4), 109.4 (C-4), 106.5 (C-1), 103.7 (OCH_2_O), 62.5 (OCH_3_), 57.7 (OCH_3_), 57.2 (C-6), 28.2 (C-5).

(**6**) ^1^H NMR (CD_3_OD, 500 MHz): *δ*_H_ 7.87 (1H, d, *J* = 9.0 Hz, H-12), 7.85 (1H, d, *J* = 9.0 Hz, H-11), 9.71 (1H, s, H-8), 8.69 (1H, s, H-13), 7.55 (1H, s, H-1), 7.03 (1H, s, H-4), 6.46 (2H, s, OCH_2_O), 4.916 (2H, m, H-6), 3.27 (2H, m, H-5), 3.98 (3H, s, OCH_3_). ^13^C NMR (CD_3_OD, 125 MHz): *δ*_C_ 152.4 (C-3), 149.0 (C-10), 148.2 (C-1), 145.6 (C-9), 145.2 (C-8), 139.4 (C-14), 134.5 (C-8a), 128.4 (C-4a), 123.0 (C-12), 122.1 (C-11), 122.0 (C-13), 121.7 (C-1a), 113.6 (C-12a), 113.0 (C-1), 112.1 (C-4), 106.1 (OCH_2_O), 57.6 (C*-*6), 56.7 (OCH_3_), 27.7 (C-5).

(**7**) ^1^H NMR (CD_3_OD, 500 MHz): *δ*_H_ 7.86 (1H, d, *J* = 9.0 Hz, H-12), 7.85 (1H, d, *J* = 9.0 Hz, H-11), 9.70 (1H, s, H-8), 8.66 (1H, s, H-13), 7.53 (1H, s, H-1), 7.01 (1H, s, H-4), 6.46 (2H, s, OCH_2_O), 4.89 (2H, m, H-6), 3.26 (2H, m, H-5), 3.97 (3H, s, OCH_3_). ^13^C NMR (CD_3_OD, 125 MHz): *δ*_C_ 152.4 (C-3), 149.0 (C-10), 148.2 (C-1), 145.6 (C-9), 145.1 (C-8), 139.2 (C-14), 134.5 (C-8a), 128.3 (C-4a), 123.0 (C-12), 122.2 (C-11), 122.0 (C-13), 121.5 (C-1a), 113.6 (C-12a), 113.0 (C-4), 112.0 (C-1), 106.1 (OCH_2_O), 57.5 (C*-*6), 56.6 (OCH_3_), 27.7 (C-5).

(**8**) ^1^H NMR (CDCl_3_, 500 MHz): *δ*_H_ 6.78 (1H, d, *J* = 8.0 Hz, H-11), 6.85 (1H, d, *J* = 8.0 Hz, H-12), 6.69 (1H, s, H-1), 6.52 (1H, s, H-4), 2.59 and 3.21 (2H, m, H-6), 2.60 and 3.12 (2H, m, H-5), 3.53 (2H, m, H-8), 3.24 (2H, m, H-13), 3.49 (1H, m, H-14). ^13^C NMR (CDCl_3_, 125 MHz): *δ*_C_ 148.5 (C-2), 148.6 (C-3), 147.9 (C-9), 145.0 (C-10), 130.1 (C-1a), 128.4 (C-12a), 127.1 (C-8a), 126.2 (C-4a), 123.3 (C-12), 110.9 (C-4), 110.1 (C-11), 108.3 (C-1), 59.8 (9-OCH_3_), 56.1 (10-OCH_3_), 56.1 (2-OCH_3_), 55.8 (3-OCH_3_), 59.2 (C-14), 54.0 (C-8), 35.9 (C-6), 28.1 (C-5).

(**9**) ^1^H NMR (DMSO-*d*_6_, 500 MHz):*δ*_H_ 8.20 (1H, d, *J =* 5.5 Hz, H-3), 7.75 (1H, d, *J =* 5.5 Hz, H-4), 7.41 (1H, s, H-5), 7.97 (1H, s, H-8), 7.00 (1H, s, H-2'), 7.03 (1H, s, H-3')。^13^C NMR (DMSO-*d*_6_, 125 MHz): *δ*_C_ 194.8 (C-1a), 164.3 (C-7'), 151.6 (C-1), 150.8 (C-6), 150.7 (C-5'), 149.9 (C-7), 146.7 (C-4'), 139.9 (C-3), 135.4 (C-4a), 133.1 (C-1'), 125.5 (C-3'), 123.4 (C-4), 113.7 (C-2'), 109.9 (C-6'), 103.0 (OCH_2_O), 102.8 (C-8), 102.4 (OCH_2_O), 101.2 (C-5), 52.1 (OCH_3_).

(**10**) ^1^H NMR (CD_3_OD, 500 MHz): *δ*_H_ 7.07 (1H, d, *J* = 7.5 Hz, H-3), 6.40 (1H, d, *J* = 7.5 Hz, H-4), 7.48 (1H, d, *J* = 8.5Hz, H-3′), 6,84 (1H, d, *J* = 8.5Hz, H-4′), 7.61 (1H, s, H-8), 6.86 (1H, s, H-5), 5.94 (2H, s, OCH_2_O), 6.04 (2H, s, OCH_2_O), 5.39 (2H, s, CH_2_), 2.52 (3H, s, CH_3_). ^13^C NMR (CD_3_OD, 125 MHz): *δ*_C_ 201.2 (C-7′), 162.7 (C-1), 152.8 (C-6), 151.4 (C-3′), 148.9 (C-2′), 148.6 (C-7), 135.4 (C-4a), 132.7 (C-6′), 131.1 (C-3), 126.6 (C-5′), 121.5 (C-8a), 117.8 (C-2′), 107.8 (C-4′), 106.9 (C-4), 105.4 (C-8), 104.2 (C-5), 102.8 (OCH_2_O), 102.5 (OCH_2_O), 47.7 (C-1a), 29.0 (C-8′).

(**11**) ^1^H NMR (DMSO-*d*_6_, 500 MHz): *δ*_H_ 6.72 (1H, d, *J* = 8.0 Hz, H-11), 6.69 (1H, d, *J* = 8.0 Hz, H-12), 6.95 (1H, s, H-1), 6,81 (1H, s, H-4), 5.99 (2H, s, OCH_2_O), 5.96 (2H, s, OCH_2_O), 3.50 (2H, m, H-8), 3.77 (2H, m, H-13), 2.80 (2H, m, H-6), 2.40 (2H, m, H-5), 1.81 (3H, s, *N-*CH_3_). ^13^C NMR (DMSO-*d*_6_, 125 MHz): *δ*_C_ 194.6 (C-14), 147.2 (C-3), 145.7 (C-2), 145.3 (C-9), 145.2 (C-10), 136.0 (C-4a), 132.6 (C-1a), 129.6 (C-12a), 125.0 (C-12), 118.2 (C-8a), 110.5 (C-4), 107.3 (C-1), 106.2 (C-11), 101.1 (OCH_2_O), 100.7 (OCH_2_O), 57.7 (C-6), 50.6 (C-8), 46.0 (C-13), 41.1 (*N-*CH_3_), 30.4 (C-5).

(**12**) ^1^H NMR (CDCl_3_, 500 MHz): *δ*_H_ 6.92 (1H, s, H-1), 6.59 (1H, s, H-4), 2.39 (2H, H-5, overlapped), 3.69 (2H, H-6, overlapped), 3.98 (2H, H-8, overlapped), 6.76 (1H, d, *J =* 7.0 Hz, H-11), 6.87 (1H, d, *J =* 7.0 Hz, H-12), 3.26 (2H, H-13, overlapped), 3.75 (3H, s, 9-OCH_3_), 3.81 (3H, s, 10-OCH_3_), 1.82 (3H, s, N-CH_3_), 5.87 (2H, s, OCH_2_O)。^13^C NMR (CDCl_3_, 125 MHz): *δ*_C_ 193.3 (C-14), 151.5 (C-10), 148.0 (C-3), 147.3 (C-9), 146.0 (C-2), 135.9 (C-14a), 132.9 (C-4a), 129.5 (C-8a), 128.5 (C-12a), 127.7 (C-12), 110.5 (C-11), 110.3 (C-4), 109.2 (C-1), 101.2 (OCH_2_O), 60.7 (9-OCH_3_), 57.5 (C-8), 55.6 (10-OCH_3_), 50.1 (C-6), 46.2 (C-13), 41.2 (*N*-CH_3_), 32.3 (C-5).

(**13**) ^1^H NMR (DMSO-*d*_6_, 500 MHz): *δ*_H_ 8.07 (1H, d, *J* = 5.5 Hz, H-3), 7.63 (1H, d, *J* = 5.5 Hz, H-4), 6.79 (1H, d, *J* = 8.0 Hz, H-2′), 6.84 (1H, d, *J* = 8.0 Hz, H-3′), 8.05 (1H, s, H-8), 7.33 (1H, s, H-5), 6.21 (2H, br s, OCH_2_O), 5.99 (2H, br s, OCH_2_O)。^13^C NMR (DMSO-*d*_6_, 125 MHz): *δ*_C_ 197.1 (C-1a), 165.4 (C-7′), 154.7 (C-1), 149.6 (C-6), 148.3 (C-6′), 147.9 (C-7), 144.3 (C-5′), 138.8 (C-3), 136.2 (C-1′), 134.4 (C-4a), 124.7 (C-6′), 122.7 (C-8a), 121.5 (C-2′), 121.4 (C-4, 106.4 (C-3′), 103.6 (C-8), 101.6 (C-5), 101.4 (C-OCH_2_O), 100.6 (C-OCH_2_O).

(**14**) ^1^H NMR (DMSO-*d*_6_, 500 MHz): *δ*_H_ 6.72 (1H, d, *J* = 8.0 Hz, H-11), 6.89 (1H, d, *J* = 8.0 Hz, H-12), 6.71 (1H, s, H-1), 6.63 (1H, s, H-4), 2.64 and 3.23 (2H, m, H-6), 2.60 and 3.11 (2H, m, H-5), 3.60 (2H, m, H-8), 3.31 (2H, m, H-13), 3.46 (1H, m, H-14). ^13^C NMR (CDCl_3_, 125 MHz): *δ*_C_ 147.5 (C-2), 147.4 (C-3), 146.9 (C-9), 146.0 (C-10), 129.3 (C-1a), 128.0 (C-12a), 127.1 (C-8a), 125.9 (C-4a), 124.0 (C-12), 109.9 (C-4), 110.8 (C-11), 108.5 (C-1), 101.3 (C-OCH_2_O), 102.1 (C-OCH_2_O), 59.2 (C-14), 54.3 (C-8), 36.22 (C-6), 27.9 (C-5).


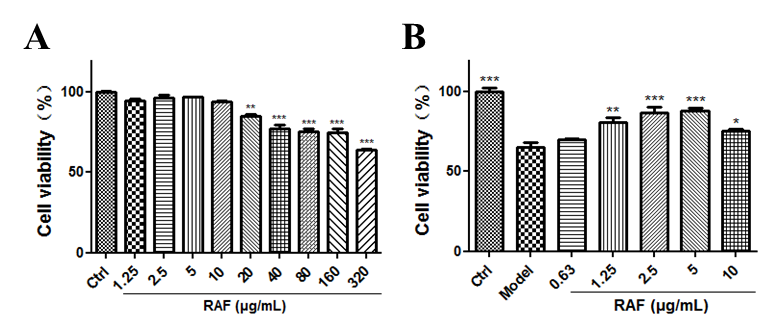


**Fig. S3** (**A**) The cytotoxicity of RAF on H9c2 cells was detected by CCK-8 assay. Data are represented as the mean ± SEM of three independent experiments. *^**^P* < 0.01, *^***^P* < 0.001, compared with the ctrl (control) group. (**B**) Detection of H9c2 cell viability after incubation in hypoxic serum-free medium for 8 h. Data are represented as the mean ± SEM of three independent experiments. *^*^P* < 0.05, *^**^P* < 0.01, **^**^P* < 0.001, compared with the Model group.
